# Supplementary material for: Efficient multi-fidelity computation of blood coagulation under flow
Source: PLoS Comput Biol. 2023 Oct 27;19(10):e1011583. doi: 10.1371/journal.pcbi.1011583 (PMC10659216; doi:10.1371/journal.pcbi.1011583)
Supplement: S1 Appendix — (PDF) [file pcbi.1011583.s001.pdf]

S1 Appendix.

Itô's Differentiation. Consider the residence time $T = T(x(t), t)$ as a function of position, $x(t)$, and time, t. Assume that the position is a stochastic process that can be modeled as a Wiener process, such as $X_t \in N(0, t)$. Then, differentiating with Itô's lemma up to second order we obtain

$$dT = \frac{\partial T}{\partial t}dt + \frac{\partial T}{\partial x}dX_t + \frac{1}{2}\frac{\partial^2 T}{\partial t^2}dt^2 + \frac{1}{2}\frac{\partial^2 T}{\partial x^2}dX_t^2 + \frac{\partial^2 T}{\partial x \partial t}dtdX_t. \tag{1}$$

Since T is the residence time, then $\frac{\partial T}{\partial t} = 1$, resulting in

$$dT = dt + \frac{\partial T}{\partial x}dX_t + \frac{1}{2}\frac{\partial^2 T}{\partial t^2}dt^2 + \frac{1}{2}\frac{\partial^2 T}{\partial x^2}dX_t^2 + \frac{\partial^2 T}{\partial x \partial t}dtdX_t. \tag{2}$$

Computing the average (i.e., the expected value, $E[-]$) and neglecting terms $O(dt^2)$ yields

$$dE[T] = dt + \frac{1}{2}\frac{\partial^2}{\partial x^2}E[T]dt, \tag{3}$$

where $E[dX_t] = dE[X_t] = 0$ and $E[dX_t^2] = dt$, (see [1]). Therefore, the total derivative of the mean residence time, $E[T] = \overline{t_R}$, can be expressed as

$$\frac{d\overline{t_R}}{dt} = 1 + \frac{1}{2}\frac{\partial^2}{\partial x^2}\overline{t_R}. \tag{4}$$

Proceeding in the same way but with the second order moment of the residence time T^2 (now considering $\partial T^2/\partial t = 2T \cdot \partial T/\partial t = 2T$), we obtain

$$\frac{d\overline{t_R^2}}{dt} = 2\overline{t_R} + \frac{1}{2}\frac{\partial^2}{\partial x^2}\overline{t_R^2}. \tag{5}$$

It is important to note that the PDE for $\overline{t_R}^2$ is not the same as the PDE for $\overline{t_R^2}$, as can be seen comparing

$$\frac{d}{dt}\overline{t_R}^2 = 2\overline{t_R}\frac{d\overline{t_R}}{dt} = 2\overline{t_R}\left(1 + \frac{1}{2}\frac{\partial^2}{\partial x^2}\overline{t_R}\right) \tag{6}$$

with eq. (5).

To calculate the spatio-temporal evolution of the variance σ_T^2, one may proceed as follows:

$$\sigma_T^2 = \overline{t_R^2} - \overline{t_R}^2 \quad \rightarrow \quad \frac{d\sigma_T^2}{dt} = \frac{d\overline{t_R^2}}{dt} - \frac{d\overline{t_R}^2}{dt}. \tag{7}$$

Substitution of equations (6) and (5) into (7) yields

$$\frac{d\sigma_T^2}{dt} = \frac{1}{2}\frac{\partial^2}{\partial x^2}\overline{t_R^2} - \frac{1}{2}\frac{\partial^2}{\partial x^2}\overline{t_R}^2 + \left(\frac{\partial\overline{t_R}}{\partial x}\right)^2 = \frac{1}{2}\frac{\partial^2}{\partial x^2}\sigma_T^2 + \left(\frac{\partial\overline{t_R}}{\partial x}\right)^2. \tag{8}$$

This expression shows that the variance grows with a source term proportional to the square of the spatial gradient of $\overline{t_R}$.

References

1. Itô K, Henry Jr P, et al. Diffusion processes and their sample paths: Reprint of the 1974 edition. Springer Science & Business Media; 1996.
