# Supplementary material for: Efficient multi-fidelity computation of blood coagulation under flow
Source: PLoS Comput Biol. 2023 Oct 27;19(10):e1011583. doi: 10.1371/journal.pcbi.1011583 (PMC10659216; doi:10.1371/journal.pcbi.1011583)
Supplement: S2 Appendix — (PDF) [file pcbi.1011583.s002.pdf]

S2 Appendix.

Multi-Fidelity Model of Third Order. Assuming that $g_i(T)$ is differentiable up to the third order, we can follow the same approach as described in the manuscript (see section Higher-order Multi-Fidelity Approximations) to derive the following expression:

$$u_i(\mathbf{x}, t) \approx g_i(\overline{t_R}) + g_i''(\overline{t_R})\frac{\sigma_T^2}{2} + g_i'''(\overline{t_R})\frac{\gamma_T}{3!} \tag{1}$$

where $\gamma = \int_{-\infty}^{\infty}(T - \overline{t_R})^3 f_T(T; \mathbf{x}, t)dT$. However, this third-order approximation introduces the third-order moment of the resident time at each fluid particle, which is denoted as $\overline{t_R^3}$. The spatio-temporal evolution of this term in an Eulerian framework can be obtained using a similar method as described in S1 Appendix for $\overline{t_R^2}$, yielding the following EDE:

$$\frac{D\overline{t_R^3}}{Dt} = 3\overline{t_R^2} + \frac{1}{2}\frac{\partial^2}{\partial x^2}\overline{t_R^3}. \tag{2}$$

Ignoring the diffusive term in (2) we obtain the *true* PDE for $\overline{t_R^3}$:

$$\frac{D\overline{t_R^3}}{Dt} = 3\overline{t_R^2}. \tag{3}$$

Consequently, the steps for the third order multi-fidelity approach are:

- **Third-order (MuFi-3)**:
  - Solve N ODEs (eq. 6 in the manuscript) to calculate $g_i(t)$ and its second and third temporal derivative $g_i''(t)$ and $g_i'''(t)$.
  - Solve three PDEs: Calculate $\overline{t_R}(\mathbf{x}, t)$ from eq. (4) in the manuscript, $\overline{t_R^2}(\mathbf{x}, t)$ from eq. (14) in the manuscript, and $\overline{t_R^3}(\mathbf{x}, t)$ from eq. (3).
  - Calculate $\sigma_T^2 = \overline{t_R^2} - \overline{t_R}^2$, $\gamma_T = \overline{t_R^3} - 3\overline{t_R}\sigma_T^2 - \overline{t_R}^3$, and map $u_i(\mathbf{x}, t) \approx g_i(\overline{t_R}) + g_i''(\overline{t_R})\frac{\sigma_T^2}{2} + g_i'''(\overline{t_R})\frac{\gamma_T}{3!}$.
