# Supplementary material for: Efficient multi-fidelity computation of blood coagulation under flow
Source: PLoS Comput Biol. 2023 Oct 27;19(10):e1011583. doi: 10.1371/journal.pcbi.1011583 (PMC10659216; doi:10.1371/journal.pcbi.1011583)
Supplement: S3 Appendix — (PDF) [file pcbi.1011583.s003.pdf]

S3 Appendix.

Womersley Inflow Boundary Condition. Considering the unidirectional motion of an incompressible flow with density ρ and kinematic viscosity ν, between two parallel flat walls separated by a distance H (as depicted in Fig 1 in the manuscript), the velocity of the fluid (v_x) is described in time, by simplifying the Navier-Stokes equation as follows:

$$\frac{\partial v_x}{\partial t} = \nu \frac{\partial^2 v_x}{\partial y^2} - \frac{1}{\rho}\frac{\partial P}{\partial x}. \tag{1}$$

In this equation, P is the pressure, while x and y are streamwise and wall-normal coordinates, respectively. Assume a pulsating pressure gradient,

$$\frac{\partial P}{\partial x} = \frac{\Delta P}{H} + \Re(A^* e^{i\omega t}), \tag{2}$$

where the operator $\Re()$ denotes the real part and ΔP is the average pressure gradient over one cycle. Then, a non-dimensional solution can be obtained for the flow with no-slip boundary conditions, i.e., $v_x(y = -H/2) = v_x(y = H/2) = 0$. This solution is characterized by velocity and length represented by U_c and H, respectively, and can be determined by applying the equation (1).

$$\tilde{v}_x = \frac{Re}{2}\Delta\tilde{P}\left(\frac{1}{4} - \tilde{y}^2\right) - \Re\left[i\frac{\tilde{A}^* Re}{\alpha^2}\left(\frac{e^{-\lambda H/2} - e^{\lambda H/2}}{e^{-\lambda H} - e^{\lambda H}}(e^{-\lambda H\tilde{y}} + e^{\lambda H\tilde{y}}) - 1\right)e^{i(\alpha^2/Re)\tilde{t}}\right], \tag{3}$$

where $\lambda = \sqrt{\frac{i\omega}{\nu}}$, $\Delta\tilde{P} = \rho U_c^2(\Delta P)$, $S_t = \omega H/U_c$, $\tilde{A}^* = A^* H/(\rho U_c^2 \Delta\tilde{P})$, $\tilde{t} = tU_c/H$ and Womersley number $\alpha = \sqrt{2\pi Re S_t}$, and Reynolds number $Re = U_c H/\nu$.
