# Supplementary material for: Efficient multi-fidelity computation of blood coagulation under flow
Source: PLoS Comput Biol. 2023 Oct 27;19(10):e1011583. doi: 10.1371/journal.pcbi.1011583 (PMC10659216; doi:10.1371/journal.pcbi.1011583)
Supplement: S4 Appendix — (PDF) [file pcbi.1011583.s004.pdf]

S4 Appendix.

Coagulation Cascade model. In this section, we report the reaction terms of the 9-species model used here. The model was presented in [1,2]. It provides a quantitative description of the intrinsic pathway kinetics, and includes the impact of PCa and the main activators of the coagulation cascade on thrombin generation. The model includes three positive feedback loops, facilitated by thrombin-activated factors XIa, Va, and VIIIa. Additionally, negative feedback mechanisms are included, wherein factors Va and VIIIa are inactivated through PCa generation, which is activated by thrombin. The model's accuracy was verified by [3], comparing it with experimentally measured kinetics of thrombin generation at various calcium concentrations.

The reaction rates of the 9 species considered in the model are provided in eqs. 1-10. The respective reaction coefficients can be found in Table A. These values were estimated through in-vitro kinetics, measured in purified systems of isolated factors [3].

$$R_{XIa} = k_{11} u_{IIa} - h_{11} u_{XIa}, \tag{1}$$

$$R_{IXa} = k_9 u_{XIa} - h_9 u_{IXa}, \tag{2}$$

$$R_{Xa} = k_{10} u_{IXa} + \overline{k_{10}} \frac{k_{89} u_{IXa} u_{VIIIa}}{h_{89} + k_a u_{PCa}} - h_{10} u_{Xa}, \tag{3}$$

$$R_{IIa} = k_2 u_{Xa} \frac{u_{II}}{u_{II} + K_{2m}} + \overline{k_2} \frac{k_{510} u_{Xa} u_{Va}}{h_{510} + k_a u_{PCa}} \frac{u_{II}}{u_{II} + \overline{K_{2m}}} - h_2 u_{IIa}, \tag{4}$$

$$R_{II} = k_2 u_{Xa} \frac{u_{II}}{u_{II} + K_{2m}} - \overline{k_2} \frac{k_{510} u_{Xa} u_{Va}}{h_{510} + k_a u_{PCa}} \frac{u_{II}}{u_{II} + \overline{K_{2m}}} - h_2 u_{IIa}, \tag{5}$$

$$R_{VIIIa} = k_8 u_{IIa} - k_a u_{PCa} \left(u_{VIIIa} + \frac{k_{89} u_{IXa} u_{VIIIa}}{h_{89} + k_a u_{PCa}} \right) - h_8 u_{VIIIa}, \tag{6}$$

$$R_{Va} = k_5 u_{IIa} - k_a u_{PCa} \left(u_{Va} + \frac{k_{510} u_{Xa} u_{Va}}{h_{510} + k_a u_{PCa}} - h_5 u_{Va} \right), \tag{7}$$

$$R_{PCa} = \frac{k_{apc1} k_p + k_{apc2} P}{k_p + P} u_{IIa} - h_{apc} u_{PCa}, \tag{8}$$

$$R_{Ia} = k_{11} u_{IIa}, \tag{9}$$

$$P = \frac{(k_{apc2} u_{IIa} - h_p k_p) + \sqrt{(k_{apc2} u_{IIa} - h_p k_p)^2 + 4 k_{apc1} k_p h_p u_{IIa}}}{2 h_p}. \tag{10}$$

Table A. Reaction Rates.

Coeff.	value	Coeff.	value	Coeff.	value
k_1	2.82 min^{-1}	k_2	2.45 min^{-1}	$\overline{k_2}$	$2 \cdot 10^3$ min^{-1}
h_2	2.3 min^{-1}	k_5	0.17 min^{-1}	h_5	0.31 min^{-1}
k_8	$1 \cdot 10^{-5}$ min^{-1}	h_8	0.31 min^{-1}	k_9	20 min^{-1}
h_9	0.2 min^{-1}	k_{10}	$3.3 \cdot 10^{-3}$ min^{-1}	$\overline{k_{10}}$	500 min^{-1}
h_{10}	1 min^{-1}	k_{11}	$1.1 \cdot 10^{-5}$ min^{-1}	h_{11}	0.2 min^{-1}
k_{89}	100 $(\text{nM} \cdot \text{min})^{-1}$	h_{89}	100 min^{-1}	k_{510}	100 $(\text{nM} \cdot \text{min})^{-1}$
h_{510}	100 min^{-1}	h_{apc}	0.1 min^{-1}	k_{apc1}	$1.4 \cdot 10^{-3}$ min^{-1}
k_{apc2}	$7 \cdot 10^{-2}$ min^{-1}	k_a	1.2 $(\text{Mn} \cdot \text{min})^{-1}$	k_p	10 nM
h_p	1 min^{-1}	K_{2m}	58 nM	$\overline{K_{2m}}$	210 nM

References

1. Zarnitsina VI, Pokhilko AV, Ataullakhanov FI. A mathematical model for the spatio-temporal dynamics of intrinsic pathway of blood coagulation. I. The model description. Thromb Res. 1996;84(4):225–236.

2. Zarnitsina VI, Ataullakhanov F, Lobanov AI, Morozova OL. Dynamics of spatially nonuniform patterning in the model of blood coagulation. Chaos. 2001;11(1):57–70.

3. Ataullakhanov FI, Pohilko AV, Sinauridze EI, Volkova RI. Calcium threshold in human plasma clotting kinetics. Thromb Res. 1994;75(4):383–394.
