# Supplementary material for: Efficient multi-fidelity computation of blood coagulation under flow
Source: PLoS Comput Biol. 2023 Oct 27;19(10):e1011583. doi: 10.1371/journal.pcbi.1011583 (PMC10659216; doi:10.1371/journal.pcbi.1011583)
Supplement: S5 Appendix — (PDF) [file pcbi.1011583.s005.pdf]

S5 Appendix.

Grid resolution study for the HiFi model. A grid resolution study was performed for the HiFi model, considering four resolutions: $\Delta x/H = 1/38$, $\Delta x/H = 1/75$, $\Delta x/H = 1/150$ and $\Delta x/H = 1/300$. The study was performed for a coagulation cascade model with $N = 3$ species [1], which is a simplification of the $N = 9$ model employed in the manuscript. The relative error was defined for the concentration of thrombin u, taking as reference thrombin concentration in the simulation with the highest resolution:

$$\bar{\varepsilon}_k(t) = \frac{1}{\Omega_{cav}} \int\int_{\Omega_{cav}} \frac{|u_k(x,y,t) - u_{300}(x,y,t)|}{u_{300}(x,y,t)} d\Omega. \quad (1)$$

Fig A shows the temporal evolution of this relative error, showing that the errors for the selected resolution, $\Delta x/H = 150$ remain below 8% for the duration of the simulation. Similar errors were obtained for the other two species considered in the model, namely factor Xa and PCa.

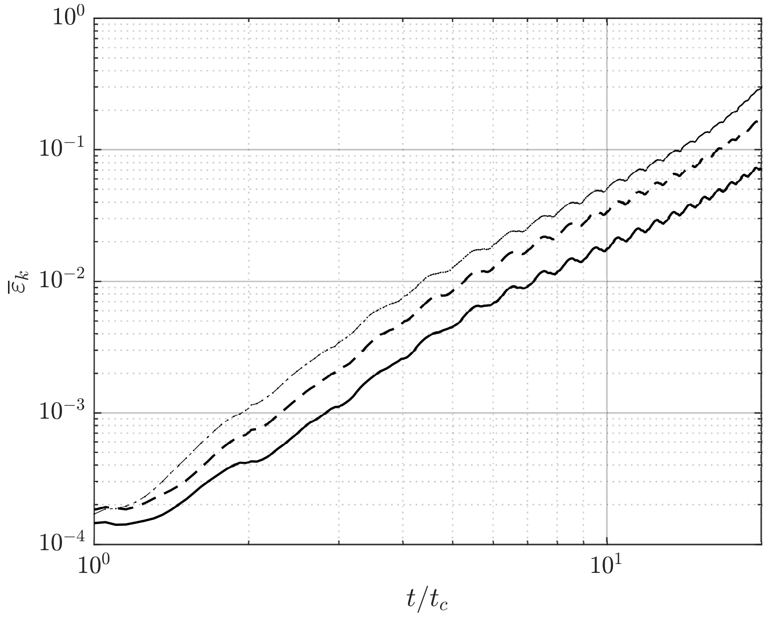

Fig A. Averaged relative error for thrombin concentration in the cavity. Each line correspond to a different spatial resolution: $\bar{\varepsilon}_{150}$ (solid), $\bar{\varepsilon}_{75}$ (dashed) and $\bar{\varepsilon}_{38}$ (dotted).

References

1. Ermakova EA, Panteleev MA, Shnol EE. Blood coagulation and propagation of autowaves in flow. Pathophysiol haemost thromb. 2005;34(2-3):135–142.
