# Supplementary material for: Efficient multi-fidelity computation of blood coagulation under flow
Source: PLoS Comput Biol. 2023 Oct 27;19(10):e1011583. doi: 10.1371/journal.pcbi.1011583 (PMC10659216; doi:10.1371/journal.pcbi.1011583)
Supplement: S6 Appendix — (PDF) [file pcbi.1011583.s006.pdf]

## S6 Appendix.

**Error maps for prothrombin and factor Xa.** For completeness, Figs A and B presents the error maps for the concentrations of prothrombin (II) and factor Xa, respectively. The errors in the concentration are plotted as a function of $\overline{t_R}$ and $\sigma_T^2$, analogous to Fig 11 in the manuscrip for the errors in the concentration of thrombin.

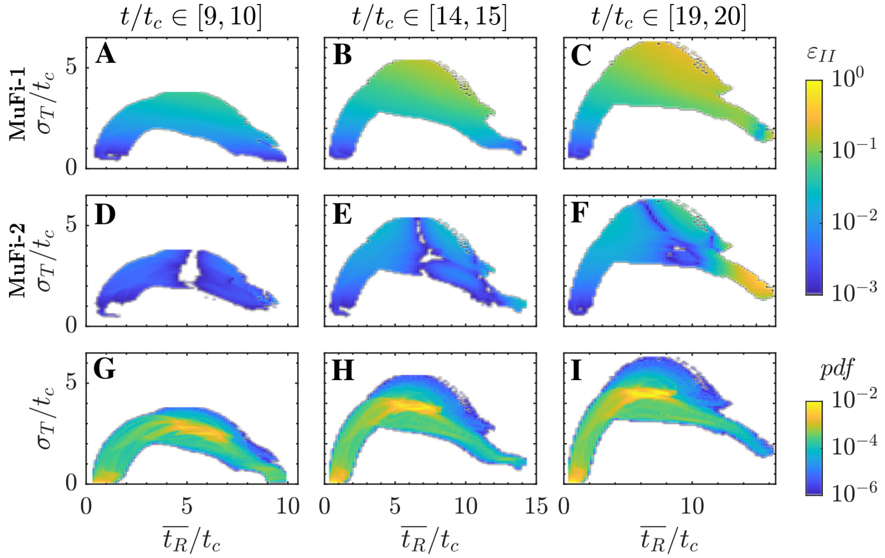


**Fig A. Error maps for prothrombin (II).** A-C: Relative error in prothrombin (II) concentration in MuFi-1, $\varepsilon^1_{II}$, as a function of residence time and its standard deviation. D-F: Same, but for MuFi-2, $\varepsilon^2_{II}$. G-I: Joint probability density function of residence time ($\overline{t_R}$) and its standard deviation ($\sigma_T$). Data for all panels is compiled inside cavity during three different cycles, as indicated on the top row. A,D,G: 10th cycle. B,E,H: 15th cycle. C,F,I: 20th cycle.

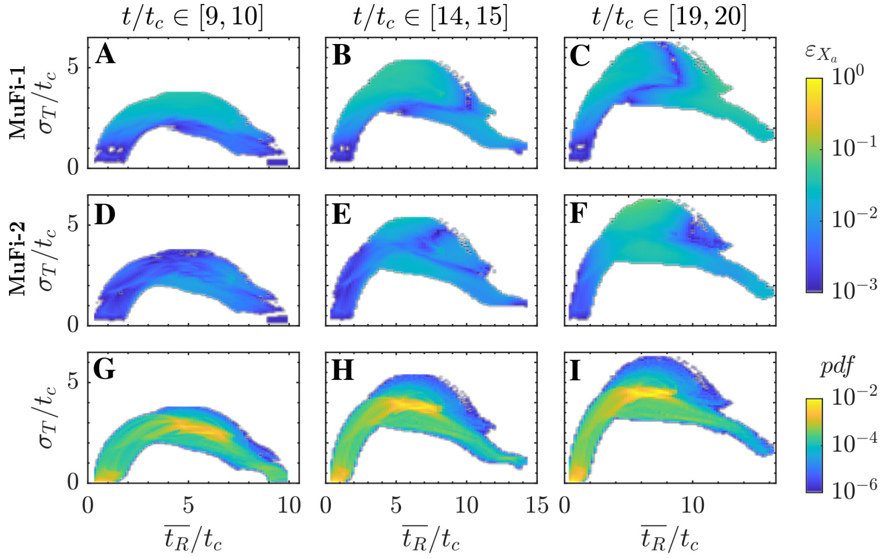


**Fig B. Error maps for factor Xa.** A-C: Relative error in factor Xa concentration in MuFi-1, $\varepsilon^1_{X_a}$, as a function of residence time and its standard deviation. D-F: Same, but for MuFi-2, $\varepsilon^2_{X_a}$. G-I: Joint probability density function of residence time ($\overline{t_R}$) and its standard deviation ($\sigma_T$). Data for all panels is compiled inside cavity during three different cycles, as indicated on the top row. A,D,G: 10th cycle. B,E,H: 15th cycle. C,F,I: 20th cycle.
