# Supplementary material for: Efficient multi-fidelity computation of blood coagulation under flow
Source: PLoS Comput Biol. 2023 Oct 27;19(10):e1011583. doi: 10.1371/journal.pcbi.1011583 (PMC10659216; doi:10.1371/journal.pcbi.1011583)
Supplement: S7 Appendix — (PDF) [file pcbi.1011583.s007.pdf]

S7 Appendix.

Averaged relative errors for $N = 3$. For completeness, Fig. 1 quantifies the overall errors of the MuFi models for a coagulation cascade model with $N = 3$ species [1]. This coagulation cascade model is a simplification of the $N = 9$ model discussed in the manuscript, accounting for thrombin, factor XIa and PCa [2]. The figure shows the time evolution of the spatially averaged relative error in the cavity (eq. 17 in the manuscript).

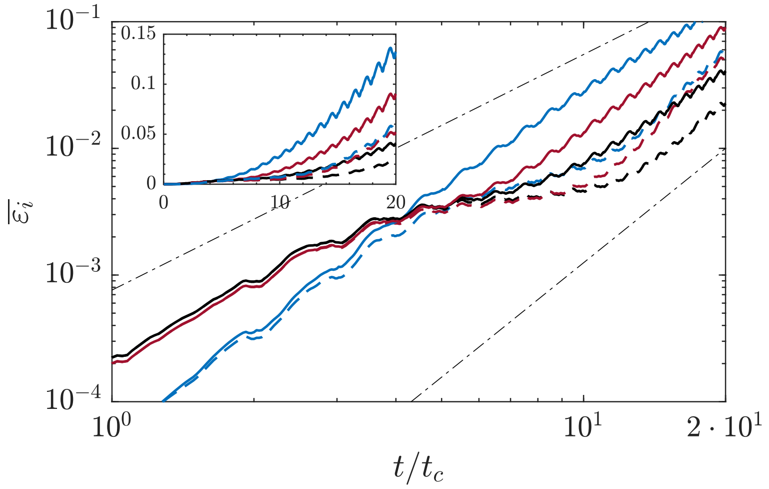

Fig 1. Averaged relative error in the cavity for $N = 3$. MuFi-1 (solid) and MuFi-2 (dashed). u_{IIa}(—), u_{Xa}(—) and u_{PCa}(—), dashed-dot lines for $\overline{\varepsilon}_i \propto (t/t_c)^2$ and $\overline{\varepsilon}_i \propto (t/t_c)^3$, top and bottom respectively.

References

1. Ermakova EA, Panteleev MA, Shnol EE. Blood coagulation and propagation of autowaves in flow. Pathophysiol haemost thromb. 2005;34(2-3):135–142.

2. Zarnitsina VI, Ataullakhanov F, Lobanov AI, Morozova OL. Dynamics of spatially nonuniform patterning in the model of blood coagulation. Chaos. 2001;11(1):57–70.
