# Supplementary material for: Efficient multi-fidelity computation of blood coagulation under flow
Source: PLoS Comput Biol. 2023 Oct 27;19(10):e1011583. doi: 10.1371/journal.pcbi.1011583 (PMC10659216; doi:10.1371/journal.pcbi.1011583)
Supplement: Attachment — Submitted filename: responses.pdf [file pcbi.1011583.s008.pdf]

Response to reviewer 1 (`PLoS Comput. Biol.-23-07-03`)

We thank this referee for the insightful comments. Here we include a point-by-point response to these comments. Changes in the revised manuscript have been highlighted in blue.

In this article, the authors propose a method to reduce the cost of thrombosis modeling. They achieve this cost reduction by reducing the number of PDEs that one has to typically solve to resolve the concentration of various species. In their proposed technique, they only solve for the residence time's PDE (and perhaps an additional PDE) and compute the concentration of various species based on the residence time through a set of ODEs that are cheap to solve.

The idea is compelling, the study is designed properly, and the manuscript is well-written. Nevertheless, there are major limitations associated with this technique. While the authors acknowledge some in the text, they are not as explicit about others. Those must be clearly mentioned in the abstract/discussion/conclusion.

We appreciate the reviewer's kind words about our work and have made an effort to address the study limitations, as detailed below.

1. First and foremost, the theoretical estimate of the cost reduction is tied to error. It is true that there is more cost saving if there are more PDEs to solve to begin with. But I am guessing the error in these computations will also be larger as N increases. To show that, note that you are effectively trying to solve $\dot{Y} = RY + LY$ where the vector Y contains all discrete solutions, matrix R is the linearized operator associated with reaction terms, and matrix L is the discrete Laplace operator. The solution to this problem is expressed in terms of eigenvalues of $R+L$. The accuracy of your approximation depends on how close eigenvalues of R are to those of $R + L$. It is true that as $||L||$ goes to zero (due to low diffusivity), the approximation becomes more accurate. But I would argue that as the number of species goes up (i.e., N), then there will be a wider range of eigenvalues in R that could get manipulated depending on whether L is dropped or not. Physically speaking that corresponds to kinetics that occur slowly (for instance the coupling associated with two species unfolds slowly), thus allowing for the diffusion process to kick in. Another supporting evidence for this argument is that the effect of modified eigenvalues on the solution accuracy grows with integration time, thus lowering the accuracy of the proposed approach with time. The reported results confirm this as they show an increase in error with time. To summarize either the authors must either acknowledge that the reported errors will likely go up as N increases or present results with an N>>3 that shows the counter.

   This is a valid concern. To address it, we repeated the numerical experiments described in the original manuscript using a 9-species system considering prothrombin (II), thrombin (IIa), fibrin (Ia), PCa, and factors XIa, IXa, Xa, VIIIa, and Va. The 3-equation system considered in the original manuscript is a simplification of the 9-equation system under the assumption that faster chemical equations are in equilibrium. We now present the MuFi models using the 9-equation system in the revised manuscript, and the 3-equation system is relegated to an Appendix. While this revision was a major overhaul, we believe it helped address this and other comments by this reviewer.

   In short, the performance of MuFi models, measured as the ability of such models to reproduce the HiFi results, was similar when tested on the 3-equation and 9-equation systems. In the case of thrombin, the MuFi-1 and MuFi-2 models yielded roughly the same spatially averaged errors with respect to HiFi after 20 integration cycles. These results are explained in detail in the revised manuscript.

Additionally, we added a paragraph in the revised discussion stating that, while the diffusive and chemical timescales are, overall, significantly different, the non-linearity of the reaction rate terms makes it difficult to rule out that diffusion became dominant in some regions of the state space, especially as the number of species increases. We also acknowledge that, while this situation does not seem likely in the light of our analysis of 3-equation and 9-equation systems, it could make it necessary to increase the MuFi order for larger N, deteriorating the MuFi speedup.

2. The second major point is that the authors never mention the actual cost reduction. Even though that is the selling point of the proposed method, they do not report how much cost reduction they obtained in reality. I understand that some people argue that it depends on the implementation etc ... but here the authors are using identical implementations for solving residence time PDE as those of the species. Hence, the cost numbers will provide an apple-to-apple comparison for the baseline and proposed methods. On the same note, the authors should mention what was N that they considered in the abstract. Reporting other values without mentioning N is meaningless.

   We now include a section reporting the cost reduction associated with solving the 9-equation system using MuFi-1 ($\approx 16X$)and MuFi-2 ($\approx 9X$) models. We also clarify in the abstract that our study is presented for $N = 9$. Please note that the advantage of MuFi models goes beyond the MuFi vs. HiFi speedup, as they allow for reusing a residence time calculation to inexpensively calculate the coagulation cascade for different models or for different values of the reaction constants within the same model.

3. The two other limitations of the study that must be mentioned, perhaps in a dedicated section are 1) the fact that BCs for all species must be identical (there is some discussion of that in the manuscript), 2) this is only suitable for cases where thrombosis modeling is one-way coupled to the Navier Stokes. Namely, if the viscosity or boundary conditions get modified due to the thrombosis, then one must solve kinetic equations coupled with those of the fluids. Hence, this method will not be practical for those settings.

   Please note that the MuFi method could still pay off in situations where boundary conditions vary for different species. Granted, it would require additional residence-time variables. Whether this approach would be efficient or not depends on multiple factors. One of them is how many groups of boundary conditions are necessary (e.g., a MuFi-1 model for a 9-eq. system with 2 sets of boundary conditions to account for the intrinsic cascade and a spatially localized release of tissue factors would still offer a $\approx 8X$ speedup). A second factor would be whether one wants to make just one simulation or M simulations, e.g., modeling the effect of Factor Xa inhibition by a direct oral anticoagulant. There are other situations like uncertainty quantification studies, etc. where MuFi models could pay off even if they required solving a significant number of residence-time-like PDEs. We revised the discussion to clarify these ideas.

   We fully agree regarding two-way coupling. We noted this limitation in the revised discussion.

4. Two minor comments: 1) line 26: did they mean tissue factor? 2) computation of residence time dates much earlier than reference 29. See for example Józsa, János, and Tamás Krámer. "Modelling residence time as advection-diffusion with zero-order reaction kinetics." Proceedings of the Hydrodynamics 2000 Conference, International Association of Hydraulic Engineering and Research. 2000. or Esmaily-Moghadam, Mahdi, Tain-Yen Hsia, and Alison

L. Marsden. "A non-discrete method for computation of residence time in fluid mechanics simulations." Physics of fluids 25.11 (2013): 110802.

Done

Response to reviewer 2 (`PLoS Comput. Biol.-23-07-03`)

We thank this referee for the insightful comments. Here we include a point-by-point response to these comments. Changes in the revised manuscript have been highlighted in blue.

It should be noted that, in order to answer one of the questions raised in the review process (namely, do the errors of the MuFi model increase with N?), we repeated the numerical experiments described in the original manuscript using a $N = 9$ species system considering prothrombin (II), thrombin (IIa), fibrin (Ia), PCa, and factors XIa, IXa, Xa, VIIIa, and Va. The $N = 3$ equation system considered in the original manuscript is a simplification of the $N = 9$ equation system under the assumption that faster chemical equations are in equilibrium. The revised version of the manuscript evaluates the MuFi models using the $N = 9$ equation system, and the 3-equation system is relegated to an Appendix.

This paper deals with the challenges associated in solving dozens number of CDR equations to model the coagulation cascade in flowing blood. Besides the computational time, and due to large Pe number, it is usually achieved by adding numerical resolution such as upwinding. Therefore, this work proposes a very nice contribution by introduce a tailorable multi-fidelity (MuFi) models to reduce the computational cost associated with the simulating of the coagulation cascade of N species in a flow. It transforms N-PDEs of the high fidelity resolution into one or two PDEs with N ordinary differential equations by changing variables between simulation time and blood residence time. The new multi-fidelity framework is tested on 2D idealized aneurysm and the comparisons between the models are discussed. Despite several assumptions mainly related to diffusivity, the framework presents a very nice contribution to the understanding of the spatial and temporal progression of coagulation species. For all that reasons, the reviewer recommends publication while answering the following questions and adding them to the revised version:

We appreciate the reviewer's kind words about our work and hope the revised manuscript will answer their questions.

1. Does the resolution of the PDE in MuFi uses the same scheme as the N PDE Hifi?

   Yes, the Cartesian mesh is the same for all the PDEs.

2. Can the authors comment on the extension 3D simulation? is it straightforward?

   Extending this methodology to three dimensions is indeed straightforward. While a 2D idealized geometry was chosen in this manuscript to facilitate a thorough validation of the methodology, our ultimate plan is to apply it to physiologically relevant 3D anatomies. Ongoing work (e.g., see upcoming conference abstract: `https://meetings.aps.org/Meeting/DFD23/Session/L06.3`) is applying MuFi models to study the coagulation cascade in 3D patient-specific left atrial models.

3. Has the initial HiFi resolution been validated before its use?

   The resolution employed to generate the velocity fields has been validated with the grid resolution study shown in table 2 in the manuscript. For the HiFi simulations, we use the same resolution, which is the standard practice in the literature (as discussed in the introduction of the manuscript). For completeness, we have added the grid resolution study for the HiFi model in the supplementary material (S5 Appendix).

4. The authors must provide a figure on the used mesh for the resolution of NS, and the PDEs.

The mesh is a Cartesian grid with constant spacing in both directions $\Delta x = \Delta y = H/150$, where H is the parent channel width. Please understand that considering the straightforward nature of this mesh and its fineness, including such a figure would not add much value. Imagine a square grid of constant size that fits 150 elements across the width of the parent channel and 750 elements across its length.

5. May the authors provide a summary table on the cost of each method to well compare and appreciate the contribution of the Mufi approaches?

   We have added a new subsection (Computational Cost) in Results, reporting the performance of MuFi-1, MuFi-2 and HiFi.

6. The resolution of NS and the PDEs are decoupled, which may affect the blood viscosity, can the authors provides some comments on this point?

   The reviewer is right. Our approach assumes that the species concentrations within the blood do not influence its properties. Instead, they are treated as passive scalars, without taking into account the mechanisms of platelet aggregation and clot formation.

   We have clarified this point as a limitation of the method in the revised Discussion section by noting that "A notable exception would be, however, applications requiring two-way coupling between flow and coagulation".

Response to reviewer 3 (`PLoS Comput. Biol.-23-07-03`)

We thank this referee for the insightful comments. Here we include a point-by-point response to these comments. Changes in the revised manuscript have been highlighted in blue.

It should be noted that, in order to answer one of the questions raised in the review process (namely, do the errors of the MuFi model increase with N?), we repeated the numerical experiments described in the original manuscript using a $N = 9$ species system considering prothrombin (II), thrombin (IIa), fibrin (Ia), PCa, and factors XIa, IXa, Xa, VIIIa, and Va. The $N = 3$ equation system considered in the original manuscript is a simplification of the $N = 9$ equation system under the assumption that faster chemical equations are in equilibrium. The revised version of the manuscript evaluates the MuFi models using the $N = 9$ equation system, and the 3-equation system is relegated to an Appendix.

The authors presented a new numerical approach for the computation of species concentration as a function of blood residence time. Their ultimate goal is to use this approach to model the coagulation cascade. The work is of high interest as the proposed approach could significantly reduce the computation time while exhibiting a rather low error. However, there are a few points that I would like to highlight.

We appreciate the reviewer's kind words about our work and hope the revised manuscript addresses the points of concern.

1. [Intro] Line 18. The authors list here many computational models for coagulation. However, as they mention earlier in the introduction, coagulation can be triggered by many factors (abnormal blood flow, hypercoagulability and vessel injury). Could the authors please provide more details about how the different models they refer to articulate around these 3 factors potentially leading to thrombus formation?

   We have added a few sentences to the introduction, as suggested by the reviewer.

2. [Intro] Line 27. As the authors mentioned, coagulation involves different time scales. Could the authors please provide a short summary of the different time scales involved? It would really help to appreciate the challenges of such numerical modelling.

   The reviewer can found the estimation of the different time scales in lines 51-59, near the end of page 4 of the revised manuscript.

3. [Methods] Line 130. The authors explain that the simplified model has no mixing, and that the reaction rate only depends on its age, which allows them to focus on residence time. Is this a strong hypothesis? If it is a common hypothesis, could the authors please refer to the appropriate literature?

   Formally, neglecting diffusion in the HiFi equations (eq. 1 of the manuscript) is not a hypothesis. It is a simplification emerging naturally from the low magnitude of the diffusion coefficient in the equations. Specifically, by analyzing and comparing the orders of magnitude of the different terms in the HiFi equations. For the characteristic time scales in medium-large vessels in the cardiovascular system, this analysis yields in a $Pe \sim 10^7$ in eq. 2. The results presented in this study strongly suggest that this simplification is valid (i.e., good agreement between HiFi and MuFi-1 model).

   Moreover, please note that we present corrections to the zero-diffusivity simplification when diffusivity is small but non-zero (the MuFi-p models), which improve the agreement with respect to the HiFi model. We are not aware of previous works in the literature analyzing the appropriateness of this simplification, not its implications for practical problems.

4. [Methods] Line 146. The authors stated that MuFi models can be significantly cheaper than the HiFi model. Could they please provide an estimate of this "cost" difference?

   We have added a new subsection (Computational Cost) in Results, reporting the performance of MuFi-1, MuFi-2 and HiFi. Briefly, our study for a coagulation model with $N = 9$ species shows that MuFi-1 is 9 times faster than HiFi, and that MuFi-2 is 16 times faster than HiFi.

5. [Methods] Line 239. The authors used a 2D idealized geometry for their computation. Could the authors comment on their choice? Does this geometry usually result in thrombosis? They also mention the left atrial appendage as an application, but this anatomical feature exhibits high velocity gradient (high velocity at the inflow and very low velocities at the tip). Have the authors tested their modeling framework for flow fields exhibiting strong velocity gradients? Also, have they tested them in more complex flow topologies (like the left ventricle for instance)?

   We choose a 2D idealized geometry in this manuscript to facilitate a thorough validation of the methodology. This geometry includes some of the features mentioned by the reviewer, like the high velocity gradient between the main channel and the cavity, and very low velocities at the distal end of the cavity, justifying the analogy with saccular aneurysms or the left atrial appendage.

   Our ultimate plan is to apply our MuFI approach to physiologically relevant 3D anatomies. Ongoing work (e.g., see upcoming conference abstract: `https://meetings.aps.org/Meeting/DFD23/Session/L06.3`) is applying MuFi models to study the coagulation cascade in 3D patient-specific left atrial models.

6. [Methods] Line 283. The authors sampled the cycle with 35 points and then linearly interpolated. Have the authors tested different sampling rates? Could they comment on the size of the sampling interval compared to the phenomena they want to model?

   Yes, various sampling rates were analyzed when computing residence time in a slightly different geometry. The results showed very good agreement between the residence time obtained from the interpolated velocity fields, and the residence time computed together with the velocity (errors smaller than 0.1%) when the sampling rate exceeded 30 frames per cycle. In any case, the velocity field used in the MuFi-1, MuFi-2, and HiFi approaches is the same, so that any errors asociated to this simplification do not impact the validation of the MuFi-1 and MuFi-2 methodologies.

7. [Results] Line 309. The authors used a symmetric waveform, when a typical systole/diastole ratio would be around 0.3/0.7. Could the authors justify their assumption?

   Our intention is to evaluate the MuFi models in a generic flow configuration representative for cardiovascular problems, not to analyze a specific anatomy or condition (see answer to question 5 above). Consequently, we use a sinusoidal waveform for the pressure gradient for simplicity (see eq. 30), resulting in the flow rate $Q(t)$ and velocity profiles shown in figure 2. We are aware that the resulting waveform is not completely physiological, and we have added a couple of sentences to the revised manuscript acknowledging this as a potential limitation (see lines 259-261 in Methodology and 490-492 in Discussion).

8. [Results] Line 342. The authors highlight the fact that the standard deviation of the residence time reaches values that can be comparable with the mean value. Could the authors please detail what are the implications of this large value of std for the multi-fidelity modeling?

The standard deviation provides an estimate of the diffusion in the problem, which is the main source of discrepancy between the MuFi approach and the HiFi model. Figure 11, and the corresponding discussion in lines 400-408, shows a clear correlation between the standard deviation of the residence time (σ_T) and the differences between MuFi and HiFi models (ε).

9. [Results] Line 380. The special distribution of species concentrations showed variable levels of agreement between the different species. Could the authors provide a possible explanation for the differences they observed between the different species?

   In the 3-species system, the evolution of the error for all species was qualitatively similar (see S7 Appendix). In the new version of the manuscript, the 9-species system shows larger differences in the agreement of HiFi and MiFi models for each species, as shown in figure 12. We believe the differences are associated to the different growth rates of each species, i.e., the r.h.s. in equation (6).

   This issues are now discussed in Results in lines 423-441, and in the discussion in line 512-513.

10. [Results] Line 381. The authors state that the MuFi-2 model accurately replicated the HiFi model up to 15 cardiac cycles. Could the authors comment on whether this time is enough to correctly capture the reactions involved in coagulation? More specifically could they comment on the ratio between this simulation time and the coagulation model parameters K?

    Figure 3 shows that, for the N=9 model and the (physiological) initial conditions used here, the thrombin concentration reaches saturation within approximately 20 cardiac cycles. Based on this observation, it can be concluded that a time span of 15 cardiac cycles provides a reasonable duration to capture the reactions involved in the coagulation cascade for this particular reaction kinetics.

    Note that 15 cycles significantly exceeds the residence times values reported by us and others in, e.g., stagnant regions in cardiac chambers and large arteries (Seo et al 2016, doi:10.1152/ajpheart.00855.2015; Arzani 2018, doi:10.1098/rsif.2018.0486; Garcia-Villalba et al, 2021, doi:10.3389/fphys.2021.596596).

    Regarding the interpretation of the K's, while the diffusive and chemical timescales are, overall, significantly different, the existence of multiple species with their own reaction rates results in different dynamics for each species, especially as the number of species increases. We added a paragraph in the revised manuscript discussing this phenomenon (see lines 541-549).

11. [Discussion] Line 437. The authors state that the reaction kinetics of these equations are much slower than the cardiac cycle. Could they please provide an estimation for this? It will help appreciate the value of their modelling approach.

    The characteristic times of the coagulation cascade kinetics are discussed in the introduction, and presented for the $N = 9$ coagulation cascade model in Figure 3.

12. [Discussion] Line 483. The average error for the MuFi-2 model is about 5% for 20 cardiac cycles. Have the authors investigated how will this error evolve as the number of cycles increases? More generally, would this model be suitable for simulation over longer time scales?

    First and foremost, note that 20 cycles significantly exceeds the residence times values reported by us and others in, e.g., stagnant regions in cardiac chambers and large arteries

(Seo et al 2016, doi:10.1152/ajpheart.00855.2015; Arzani 2018, doi:10.1098/rsif.2018.0486; Garcia-Villalba et al, 2021, doi:10.3389/fphys.2021.596596).

As outlined in S2 Appendix, the third-order multi-fidelity model (MuFi-3) can be described in terms of the MuFi-2 formulation plus an additional term proportional to the third derivative of g_i''' and the third moment centered around the mean residence time, denoted as γ_T. This could be used to improve the performance of the MuFi model for longer times.

13. [Discussion] Line 531. The authors describe other approaches to obtain residence time, from in vivo or in vitro models. Have they thought about any way to confront their numerical approach with in vitro experimental data for instance?

    This issue is now addressed in the new version of the manuscript, lines 569-571.

14. Minor comments

    Figure 3. Could the authors please add the species associated with u1-3 in the legend of the figure?

    Done

15. Figure 5. The color scale for the plot of the mean residence time tends to be hard to read. Could the authors please use a linear color scale?

    Done

16. Figure 6. Could the authors add a schematic for the locations of the 3 data points? They refer to Figure 5A, but having all the information in Figure 6 would help the reader. This comment can also apply to Fig 9.

    Done

17. Figures. Overall, the tables of figures are sometimes difficult to read. Having the type of model included in the figure would help.

    Done

18. Line 229. Please add a space before 14.

    Done

19. Line 412. Please add a space before the bracket.

    Done
